# Fexofenadine: review of safety, efficacy and unmet needs in children with allergic rhinitis

**DOI:** 10.1186/s13223-021-00614-6

**Published:** 2021-11-02

**Authors:** Eli O. Meltzer, Nelson Augusto Rosario, Hugo Van Bever, Luiz Lucio

**Affiliations:** 1 Department of Pediatrics, Division of Allergy and Immunology, University of California, La Jolla, San Diego, CA USA; 2 Departamento de Pediatria, Universidade Federal Do Parana, Curitiba, PR Brazil; 3 Department of Pediatrics, Division of Rheumatology, Immunology, Allergy, Yong Loo Lin School of Medicine, National University of Singapore, Singapore, Singapore; 4 Medical Department, Sanofi Consumer Healthcare, AI, Traira 456, Santana de Parnaiba-SP, Brazil, São Paulo, 06540 365 Brazil

**Keywords:** Allergic rhinitis, Fexofenadine, Pediatrics, Quality of life, Second-generation antihistamines, Sedative effect

## Abstract

Allergic rhinitis (AR) is the most common undiagnosed chronic condition in children. Moderate/severe AR symptoms significantly impair quality of life, and cause sleep disruption, absenteeism and decreased productivity. Additionally, untreated AR predisposes children to asthma and other chronic conditions. Although intranasal corticosteroids are the most effective pharmacologic treatment for AR, oral antihistamines are often preferred. First-generation antihistamines may be chosen to relieve AR symptoms as they are inexpensive and widely available; however, they cause sedative and cardiovascular negative effects due to poor receptor selectivity. Therefore, second-generation antihistamines were developed to reduce adverse effects while retaining efficacy. There are fewer clinical trials in children than adults, therefore, efficacy and safety data is limited, particularly in children under 6 years, highlighting the need to generate these data in young children with AR. Fexofenadine, a highly selective second-generation antihistamine, effectively alleviates symptoms of AR, is non-sedating due to decreased blood–brain barrier permeability, and is devoid of cardiovascular side effects. Importantly, fexofenadine relieves the ocular symptoms of allergic conjunctivitis, which occur concomitantly with AR, improving quality of life. Overall, fexofenadine displays a favorable safety profile and results in greater treatment satisfaction in children compared with other second-generation antihistamines. This review aimed to evaluate and compare the safety and efficacy of fexofenadine with other available first- and second-generation antihistamines in children with AR.

**Supplementary Information:**

The online version contains supplementary material available at 10.1186/s13223-021-00614-6.

## Introduction

**Fig. 1:**
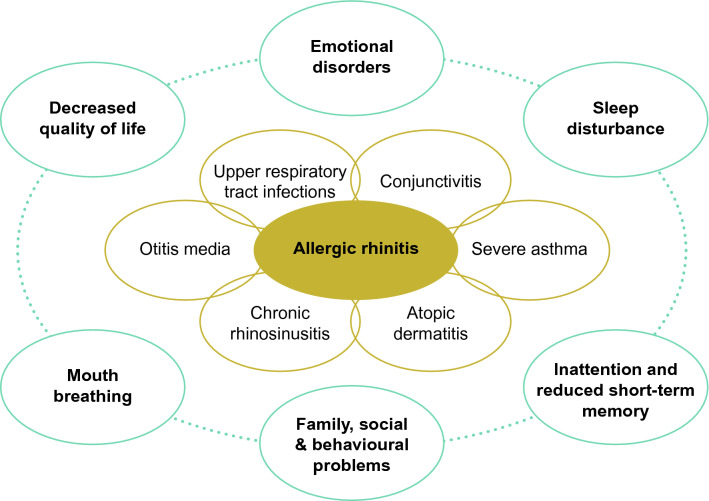
Complications and consequences of allergic rhinitis

Allergic rhinitis (AR), a Type 1, immunoglobulin E (IgE)-mediated, hypersensitivity reaction, is the most common chronic condition in children, afflicting up to 40% of the pediatric population worldwide [1, 2]. Cross-linking of IgE on mast cells occurs upon allergen exposure triggering the release of several mediators, including histamine, leukotrienes and cytokines, which lead to the classic symptoms of AR [3]. AR has previously been categorized by type of allergen, with perennial AR associated with indoor allergens such as house dust mites, and seasonal AR associated with outdoor allergens, such as pollen. However, this classification is not reliable since most patients are polysensitized, causing multi-season, or, in some locations, perennial problems, as indoor and outdoor allergen levels differ throughout the year and therefore affect sufferers episodically or year round [4]. Attributed to this, the Rhinitis 2020: A practice parameter update recommended that AR be classified according to severity (mild or moderate/severe), frequency (intermittent [< 4 days/week or < 4 consecutive weeks at a time] or persistent [≥ 4 days/week and ≥ 4 consecutive weeks/year]), and environmental exposure [5]. Mild AR symptoms do not interfere with quality of life (QoL), and have no impact on daily activities, work or school performance, leisure activities and sleep; however, moderate/severe symptoms can be troublesome and negatively impact any or all of these aspects of daily life [5]. They can significantly impact physical, social, emotional and mental aspects of life. AR was previously thought to be a localized disorder; however, AR may be present as a component of systemic airway disease, and has been associated with other comorbidities such as asthma, chronic rhinosinusitis, with or without nasal polyps, otitis media, allergic conjunctivitis and atopic dermatitis (Fig. 1) [2, 3, 6, 7].

**Fig. 2:**
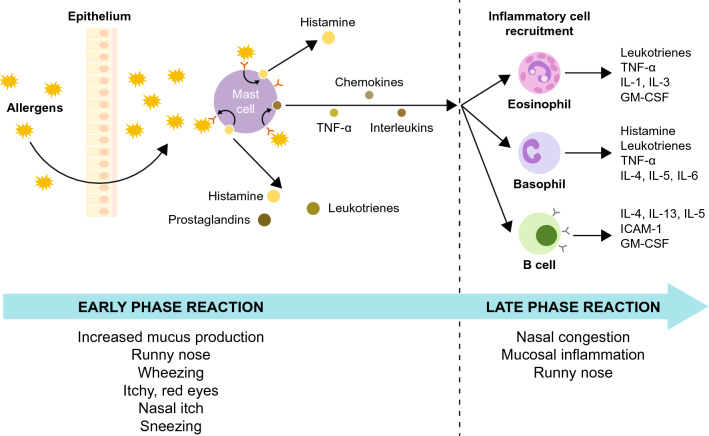
Pathophysiology of allergic rhinitis. In the early phase reaction, allergen exposure results in mast cell degranulation and the release of mediators such as histamine, which produce the typical symptoms of AR, including sneezing and mucus production. In the late phase reaction, the release of cytokines and chemokines from mast cells leads to recruitment of inflammatory cells such as basophils, lymphocytes and eosinophils and the release of mediators such as histamine and leukotrienes, which results in sustained nasal congestion and inflammation B cell, B lymphocyte; GM-CSF, granulocyte macrophage colony-stimulating factor; ICAM-1, intercellular adhesion molecule 1; IL-1, interleukin 1; IL-3, interleukin 3; IL-4, interleukin 4; IL-5, interleukin 5; IL-6, interleukin 6; IL-13, interleukin 13; TNF-α, tumor necrosis factor alpha

Classic symptoms of AR, include increased sneezing, mucus secretion, nasal itch and nasal congestion (blockage), and are often accompanied by ocular symptoms such as itchy, red eyes and increased blinking and lacrimation (Fig. 2) [8]. Given the important role of histamine in the allergic response and their ease of use, oral antihistamines are often the first-line of pharmacological treatment for the management of AR symptoms [9]. Oral antihistamines are categorized into first-generation and second-generation, primarily based on their associated adverse effects [10]. Although effective at relieving many AR symptoms, first-generation antihistamines display poor receptor selectivity, therefore, frequently induce sedative, cardiovascular and/or anticholinergic effects [11, 12]. As such, the therapeutic ratio of first-generation antihistamines is problematic, particularly in children, who can be unknowingly compromised by these adverse effects [11]. Second-generation antihistamines were subsequently developed with less ability to cross the blood–brain barrier, less effect on cardiac ion channels and better receptor selectivity, therefore avoiding the unwanted effects associated with first-generation antihistamines [11, 13, 14]. Second-generation antihistamines are clinically effective histamine (H_1_)-receptor inverse agonists and are widely used in children due to their low propensity to induce sedation, high degree of cardiac safety and low capacity to bind to cholinergic receptors [11, 14]. This review evaluates the safety and efficacy of fexofenadine, a second-generation oral antihistamine, in children with AR. Clinical trials with fexofenadine included children from 6 months up to 11 years [13, 15–22]. However, due to the limited feasibility of specific clinical trials in a pediatric population, particularly in younger children, a limited number of results from clinical studies in adults have been included for comparison [23–30].

### The burden of allergic rhinitis

QoL is rarely impacted by mild AR symptoms; however, the majority of children, aged 12–17 years, experience moderate (50%) or severe (38%) symptoms, with sleep disorders experienced by approximately 83% of children with persistent moderate/severe AR [3, 23, 31]. Nasal congestion, the primary cause of sleep disturbance, habitual snoring and obstructive sleep apnea syndrome have been documented in children with AR and often result in daytime fatigue, irritability, tiredness, inattention, reduced short-term memory and behavioral problems, significantly affecting learning and social activities [6, 32]. Since seasonal AR especially occurs in Spring and Autumn, the majority of children suffer from AR symptoms during the school year, with AR-related sleep disturbances resulting in tiredness and distraction in the class room, termed presenteeism, leading to decreased productivity and impaired learning [2, 6, 32]. One study found that, during a typical allergy season, 91% of children with seasonal AR reported suffering decreased productivity at school at least one day a month and approximately 54% experienced 6–20 days of diminished productivity; they were subsequently estimated to have lost an average of 10.2 days to AR symptom-related decreased productivity per allergy month [23]. AR symptoms can also lead to frequent absenteeism, with an estimated two million school days lost annually in the US [25, 32, 33]. In addition, AR and associated allergic conjunctivitis can lead to emotional disorders linked to embarrassment, shame, poor self-esteem, depression, and family problems caused by parental anxiety, hostility and overprotection [32]. AR is also a predisposing factor for asthma development and, when poorly managed, can increase the risk for severe asthma symptoms [34].

### Treatment

The US Rhinitis practice parameter update supports a stepwise approach to treatment, which should consider relative effectiveness, onset of action, potential adverse effects, patient preference, cost, symptom severity and the presence of either intermittent or persistent AR [5].

AR symptom management in children is similar to that of adults, involving allergen avoidance, pharmacological treatments and allergen immunotherapy. Allergen avoidance is important, but major effectiveness by this intervention is often unachievable [2, 3, 35]. There are multiple pharmacological options available for AR symptom management. Systemic corticosteroids are only recommended for short term treatment of severe AR due to local and systemic side effects; however, intranasal corticosteroids are the most effective treatment for AR, as they minimize the adverse pathophysiologic consequences of allergic inflammation. Oral leukotriene receptor antagonists (e.g. montelukast) are only modestly effective for AR and as such, are not routinely offered as therapy. Intranasal decongestants can reduce nasal airway obstruction, but use should be limited to 3–5 days to avoid tachyphylaxis (sometimes referred to as sub-sensitivity) and rebound congestion [1, 3]. Oral decongestants can be recommended, with pseudoephedrine the most effective monotherapy, and can also be taken in combination with oral antihistamines; however, they are generally avoided in young children due to the narrow margin of safety between therapeutic and toxic doses [1, 3, 36]. Oral antihistamines are the most used treatment option in children due to this being the easiest and, therefore, the preferred route of administration [37]. Thus, both intranasal corticosteroids and oral antihistamines are considered the first-line of pharmacological treatment for children with AR [5]. Allergen immunotherapy is quite effective and should be considered for those suffering from moderate/severe AR whose symptoms are not controlled by allergen avoidance and pharmacotherapy, and in those with comorbidities such as asthma.

Because therapeutic choices are often patient preference dependent, oral antihistamines are considered a mainstay treatment for alleviating AR symptoms in children (Table 1) [7, 8, 10].

**Table 1:** Antihistamines categorized by generation

First-generation	antazoline, chlorphenamine, clemastine, cyproheptadine, dimetindene, diphenhydramine, hydroxyzine, ketotifen, promethazine, triprolidine
Second-generation	acrivastine, azelastine, bilastine, cetirizine, desloratadine (metabolite of loratadine), ebastine, emedastine, fexofenadine (metabolite of terfenadine), levocetirizine (enantiomer of cetirizine), loratadine, mizolastine, olopatadine, rupatadine

Second-generation antihistamines are favored as the first-line treatment option for the management of AR, since first-generation antihistamines are associated with various potential adverse effects [3, 5, 7, 8, 35, 38]. When taken regularly, second-generation oral antihistamines effectively reduce sneezing, itching and rhinorrhea [3]. They are generally less effective in the treatment of nasal congestion [7]. Importantly for a chronic disease, long term use of second-generation antihistamines does not cause tachyphylaxis.

### Antihistamines

Beginning in the 1940s, antihistamines were shown to be an effective pharmacological treatment option for AR, following the understanding that histamine and its receptors play an important role in the development of allergic symptoms [39]. It is widely recognized that antihistamines act as competitive antagonists of H_1_-receptors to prevent the binding of circulating histamine; however, some antihistamines also act as inverse agonists [40]. Antihistamines that act as inverse agonists are not structurally related to histamine and instead, bind to different sites, stabilizing the inactive form of H_1_-receptors and therefore suppressing the constitutive activity of the H_1_-receptor [41].

Since medical care is rarely sought for mild AR symptoms, many sufferers self-prescribe with over-the-counter (OTC) medications to manage symptoms [3]. As a result, OTC first-generation antihistamines, such as chlorpheniramine and diphenhydramine, are still widely used as they are inexpensive and effectively alleviate AR symptoms [8]. However, the associated side effects, such as cognitive and psychomotor impairment, can further add to the burden of AR [1, 2, 6, 8, 41, 42]. Although effective, the therapeutic ratio for first-generation antihistamines is poor as the associated anticholinergic activity can result in accidental overdosing in children, even at recommended doses [1, 2]. Despite the potential adverse effects, first-generation antihistamines are unfortunately still commonly recommended and used in children with AR [1].

Since the 1980s, second-generation antihistamines, including acrivastine, azelastine, bilastine, cetirizine, desloratadine, fexofenadine, levocetirizine and loratadine, have been developed to reduce the potential important central nervous system (CNS) depressant/sedative side effects while displaying similar efficacy to that of first-generation antihistamines (Table 2) [1, 2, 42–45]. Azelastine, an intranasal antihistamine, has beneficial local mucosal effects, with rapid onset of therapeutic effect and a good safety profile [46]. In addition to being prescribed as a monotherapy, azelastine has been developed in combination with intranasal corticosteroids to exploit the properties of both classes of these agents [45]. Second-generation antihistamines are being constantly developed to enhance selectivity and potency, whilst reducing potential side effects. Bilastine, recently approved for use in children aged 6–11 years with AR, displays similar efficacy to cetirizine and a similar safety profile to fexofenadine, but with longer duration of action [47]. Furthermore, unlike many other available antihistamines, rupatadine (1 mg/mL oral solution), a dual antagonist (H_1_ and platelet aggregating factor), has proven safe and effective in children > 2 years with AR [48], demonstrating greater potency than fexofenadine [49].

**Table 2:** Side effects of the different generations of antihistamines

Class	Central nervous system	Cardiovascular	Toxic high dose
First-generation	agitation, confusion, dystonia, dyskinesia, hallucinations, headache impairment in coordination, learning, memory, psychomotor and sensorimotor functions, and sedation	dose-dependent sinus tachycardia, reflex tachycardia, atrial refractory period prolongation and supraventricular arrhythmias	severe CNS and cardiac side effects, may lead to death unless treated
Second-generation	variable (such as sedation with cetirizine) Minimal or no side effects	no side effects	no severe side effects or deaths reported

CNS, central nervous system

### Fexofenadine

Fexofenadine is approved as an oral tablet and a liquid suspension for the relief of symptoms of AR (≥ 2 years) or urticaria (≥ 6 months). The recommended dose for children, aged ≥ 12 years, and adults is 60 mg twice daily, or 120 mg and 180 mg once daily [50]. The oral suspension of fexofenadine (30 mg, twice daily) is recommended for the treatment of seasonal AR in children aged 2–11 years. For children > 6 years, an oral tablet of fexofenadine is available [18, 50, 51].

Fexofenadine is a highly selective H_1_-receptor antagonist with less affinity for cholinergic or α-adrenergic receptors and therefore displays negligible adverse effects compared with first-generation antihistamines [14, 19, 20, 22, 40, 52]. Additionally, lack of CNS penetration means that fexofenadine does not induce sedation, and does not impair concentration, memory or performance [19, 26, 28, 41].

In adults, fexofenadine is rapidly absorbed, reaching peak plasma concentrations 1 h after administration, with a single dose of 130 mg achieving maximum histamine inhibition 1–2 h after administration [53]. Similarly, 30 mg or 60 mg of fexofenadine in children has been shown to suppress histamine-induced wheal and flare within 1 to 2 h of administration [17].

To determine the dose of fexofenadine in children that yielded a similar effect to adults receiving 60 mg twice a day, fexofenadine was administered to children aged 6 months to 2 years (15–30 mg), 2–5 years (30 mg) and 6–12 years (30 mg), and the effect of weight, age, gender, race, height and body surface area on the pharmacokinetic behavior of fexofenadine was analyzed [54]. Overall, oral clearance was reduced by 61% in children aged 6 months to 2 years, 36% in children aged 2–5 years and 44% in children aged 6–12 years. In children aged 6 months to 2 years, clearance was 56% and 72% lower than adults when stratified by weight > 10.5 kg and ≤ 10.5 kg, respectively. The study suggested that 30 mg fexofenadine can be administered twice a day in children 1–12 years, with only a small group of children < 10.5 kg, requiring a reduced dose of 15 mg [54]. In contrast, another study assessing the pharmacokinetic behavior of fexofenadine in Japanese children aged 6 months to 2 years (15 mg, twice a day) determined that dose need only be adjusted based on age, and that body weight is of minor importance [16]. An additional study confirmed that children aged 2–5 years given a single dose of 30 mg fexofenadine had similar exposure to 30 mg and 60 mg given to children aged 6–11 years and adults respectively [18]. There are however, few studies examining the influence of demographics on the pharmacokinetics of fexofenadine in children under 12 years, and none in children under 6 months. Pharmacokinetic data for fexofenadine in children, by age group, is shown in Table 3.

**Table 3:** Pharmacokinetics of fexofenadine in children

Fexofenadine dose and frequency	Age, years	Number of patients	Mean maximum plasma concentration, ng/mL	Mean area under the plasma concentration curve, ng hour/mL	Drug clearance	References
15 mg BID	Infants < 2	55	130^‡^	**–**	15.6 L/h	[16]
30 mg BID	> 2– < 7	80	157^‡^	**–**	29.9 L/h	[16]
30 mg, single dose	2–5	50	224^*^	898	–	[18]
30 mg, single dose	6–12	14	178 ± 22^†^	1090 ± 125	14.4 ± 2.0 ml/min/kg	[17]
60 mg, single dose	6–12	14	286 ± 34^†^	1892 ± 129	18.4 ± 2.4 ml/min/kg	[17]

BID, twice daily; QD, once daily

^*^24 h post dose

^†^2.4 ± 0.2 h post dose

^‡^Day 8, 3 h and 3–9 h post dose

### Safety profile

The safety of fexofenadine has been investigated extensively in adults and school age children and some clinical trials have evaluated the safety of fexofenadine in children under 6 years**.** The established therapeutic range for fexofenadine in adults and children over 12 years is 20–240 mg [55, 56]. No dose-related trends in adverse effects have been noted with varying doses of oral fexofenadine (15, 30 and 60 mg, twice daily) in children, with fexofenadine displaying a similar safety profile to that of placebo [1, 15, 22]. Fexofenadine was well-tolerated and displayed a good safety profile in children with AR, aged 6 months to 2 years, at doses of 15 and 30 mg twice daily [13, 21]. Several other studies have confirmed that daily doses of 30–60 mg of fexofenadine are safe and well-tolerated in children as young as 2 years, with a similar safety profile to that seen with adults [12, 19, 22]. The incidence of fexofenadine-related adverse events was also similar to that observed with placebo for children aged 2–5. An overview of fexofenadine-related adverse events can be found in Additional file 1: Table S1 [51]. Headache was commonly reported by AR sufferers receiving fexofenadine treatment during clinical trials, occurring at a similar incidence to placebo [12, 57]. Additionally, long-term studies in healthy volunteers aged 12–65 years demonstrated that fexofenadine is safe and well tolerated when doses up to 240 mg are given once a day for up to 12 months [24].

### Second-generation antihistamines display reduced CNS effects

Second-generation antihistamines were developed to minimize the central and peripheral side effects observed with first-generation antihistamines [58]. As a result of careful assessments, second-generation antihistamines are documented to be less sedating than first-generation antihistamines. For example, first-generation antihistamines such as diphenhydramine and hydroxyzine, cause undesirable sedative effects which impact concentration, learning ability, attention, memory and coordination [27]. However, studies have also shown that sedative properties differ even within second-generation antihistamines. Fexofenadine has been shown to occupy none of the H_1_-receptors in cerebral cortex, compared with ~ 20–50% occupancy for cetirizine, indicating that cetirizine produces a greater sedative effect than fexofenadine even though they are both considered second-generation antihistamines. Additionally, both desloratadine and loratadine display anticholinergic activity whereas fexofenadine, cetirizine and levocetirizine are highly specific for H_1_-receptors [10]. As a result, fexofenadine can be considered one of the least sedating and most receptor specific second-generation antihistamines compared with other antihistamines [27, 59].

**Fig. 3:**
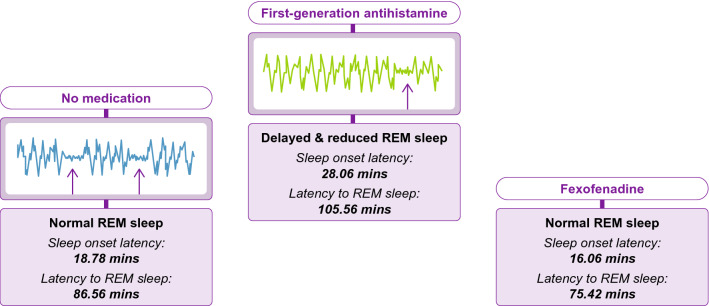
The effects of first-generation antihistamines on the sleep/wake cycle. REM, rapid eye movement. Data from Church et al. *Allergy*. 2010 Apr; 65(4):459–466 and Boyle et al. Curr Med Res Opin; 22:1343–1351

Several clinical studies have objectively measured the sedative properties of fexofenadine in children, and the subsequent impact on QoL, and found that fexofenadine improved all disease measures [2, 25]. The effect of fexofenadine on sleep has been measured using electroencephalography and polysomnography to analyze brain chemistry and overnight sleep, respectively. The multiple sleep latency test has also been used to assess daytime sleepiness [26]. As a result, it has been shown that second-generation antihistamines, such as fexofenadine, do not have the same impact on sedation as first-generation antihistamines [41, 60]. For instance, first-generation antihistamines, such as chlorpheniramine, are known to increase sleep latencies to sleep onset and rapid eye movement (REM) sleep, and reduce the duration of REM sleep (Fig. 3). No such sleep latencies have been noted with fexofenadine and other second-generation oral antihistamines [14, 61]. When administered at night, chlorpheniramine-related sleep disturbances, along with a long drug half-life, can result in a ‘hangover’ effect the following morning [14, 61]. In the past, first-generation antihistamines were recommended for evening use in children, assuming their sedative properties would allow for a higher quality of sleep. However, the resulting disruption in circadian sleep–wake rhythmicity and subsequent ‘antihistamine hangover’ negatively impacts attention, working memory, learning and overall school performance the following day [42, 61, 62]. Unlike chlorpheniramine, night-time use of fexofenadine does not disrupt sleep and is not associated with an ‘antihistamine hangover’ and, therefore, is not detrimental to psychomotor or cognitive performance the next day [14, 27, 61]. Despite the contradictory evidence, first-generation antihistamines are still commonly prescribed for use in children with AR at night time [1].

Fexofenadine displays no sedative properties in children and in children aged 6–11 years, even when used at higher than recommended doses [2, 22]. Additionally, fexofenadine was not associated with any objective/subjective performance, or cognitive/academic impairment [22]. Importantly, multiple studies have compared the CNS effects of several second-generation antihistamines, including cetirizine, loratadine, levocetirizine and fexofenadine. Although cetirizine produced modest sedative effects, and both levocetirizine and desloratadine produced mild sedative effects, fexofenadine had no adverse effect. These data suggest that, of the oral antihistamines available, fexofenadine is the only one that does not impair cognitive and psychomotor function, though more head-to-head trials are needed to confirm this [41, 42, 44, 63]. Overall, a limited number of antihistamines are devoid of sedative effects; however, since fexofenadine displays minimal CNS infiltration, and therefore does not appear to impair cognitive and psychomotor performance, it is considered one of the few truly non-sedating antihistamines, regardless of dose [64, 65].

### Cardiac safety of second-generation antihistamines

Although less common, cardiac toxicity is a potentially severe side effect of both first and second-generation antihistamines [58]. For example, second-generation antihistamines, terfenadine and astemizole, were withdrawn from the market in the 1990’s as they were shown to block cardiac potassium channels at relatively low concentrations, causing prolongation of the QT interval [27, 66–68]. Because of this concern, the electrocardiographic effects of fexofenadine, an active metabolite of terfenadine, were extensively studied in adults and no significant effect on heart rate, PR interval (time from the onset of the P wave to the start of the QRS complex), QRS width, QT interval or QTc (corrected QT interval) were found. In healthy adult volunteers, single doses of up to 800 mg and multiple doses up to 690 mg twice a day have shown a cardiac safety profile similar to placebo [27–29, 69]. Additionally, multiple studies have shown that fexofenadine has no dose-related effect on QTc, with cardiovascular safety established up to doses of 1380 mg [22, 29].

Notably, in 1999 there was one case report of *torsade de pointes* causally associated with exposure to fexofenadine; however, taking into account the patients’ existing left ventricular hypertrophy, age, arterial hypertension and cessation of hypertensive therapy, conclusions on the cardiac safety of fexofenadine could not be drawn [30, 66, 69, 70]. In response, the safety of 180 mg fexofenadine was assessed in a further 432 patients with urticaria and no events of ventricular tachycardia or electrocardiogram changes of QTc prolongation were reported [30]. In addition, the pooled analysis of 2100 patients also showed that fexofenadine does not increase QTc, even when administered at ten-fold higher than the recommended dose [30].

There have been limited studies on the cardiac safety of fexofenadine in children; however, clinical findings in adults may also be applicable to children. Two such studies assessing the safety and tolerability of fexofenadine, 15 and 30 mg, twice daily in children 6 months to 2 years showed an absence of potential cardiovascular effects even with a two-fold dose, with vital signs, electrocardiographic results and physical examination findings similar to placebo [13]. Similarly, no prolongation in QT interval was observed and no clinically relevant changes from baseline were found at study end in laboratory measures, vital signs, or physical examination findings in children aged 2–5 years taking 30 mg fexofenadine twice daily, and in children aged 6–11 years taking either 15, 30 or 60 mg of fexofenadine twice daily [19, 22].

### Efficacy

An overview of studies assessing the efficacy of fexofenadine in children can be found in Additional file 1: Table S2. For children aged 2–11 years, the recommended dose of fexofenadine to relieve AR symptoms is 30 mg twice daily. The use of 30 mg for a wide age range is supported by the fact that 30 mg produces exposures similar to those seen with the 60 mg dose in adults [54]. This recommended dose has demonstrated efficacy and safety in different clinical trials and might offer distinct advantages compared with other antihistamines with respect to symptom management [12–14, 27, 71].

Several studies, using total symptom score (TSS) and individual symptoms scores to assess the efficacy of fexofenadine in children aged 6–11 years with seasonal AR, showed that twice daily dosing with 30 mg fexofenadine effectively reduced all symptoms of AR, including nasal congestion, and symptom reduction was maintained throughout the 2-week study period [1, 15, 21, 50]. Importantly, although antihistamines are generally considered ineffective at reducing nasal congestion, fexofenadine (30 mg) has been shown to significantly relieve all symptoms of seasonal AR, including nasal congestion. By targeting the vascular mediators responsible for nasal congestion, fexofenadine displays broader activity than other antihistamines [15].

To assess the effectiveness of AR treatment, clinical trials should measure the change in reflective total nasal symptom score (TNSS), from baseline to end of study (2 weeks for seasonal and 4 weeks for perennial AR) however, few studies have analyzed subjective efficacy endpoints, such as TNSS, in children with AR [15, 72]. Nevertheless, overall efficacy satisfaction for AR symptom relief was higher for second-generation antihistamines compared with first-generation antihistamines, with fexofenadine highly rated in children aged 1–12 years [61]. Furthermore, along with providing relief of seasonal AR symptoms, fexofenadine has also been shown to alleviate symptoms of perennial AR [65].

## Unmet needs and future research on fexofenadine in children

Although intranasal corticosteroids are the most effective treatment option for the relief of AR symptoms, oral antihistamines are more commonly used due to the patient’s preference for the oral route of administration [37, 38, 73]. Even though antihistamines are the most commonly used medication, the number of clinical trials assessing the safety, efficacy and the influence of demographics on the pharmacokinetics of antihistamines in the pediatric population is limited and often conflicting. This lack of data in children with AR, particularly those under 6 years, limits the ability to perform comprehensive meta-analysis and systematic reviews. This is further compounded by the fact that clinical trials in young children with AR are particularly challenging since young children are usually unaware of their symptoms and generally less able to define and quantify them. Hence, the studies require a caregiver to report the child’s symptoms and changes in their magnitude. The second hand nature of this situation often blunts the ability to collect subjective data from patients of this age. In response, we have included data from a small number of clinical studies in adults; however, these findings may not be comparable. Furthermore, AR is rarely found in isolation. The consideration of potentially confounding symptoms and diagnoses, such as upper respiratory tract infections, and the fact that AR is frequently not diagnosed by pediatricians even though it may present in infancy, contribute to under diagnosis and under treatment [74]. In adults and children over 12 years, research has focused on the classical nasal symptoms of AR, such as nasal congestion; however, a high percentage of patients with AR also experience the ocular symptoms of allergic conjunctivitis. Though these ocular symptoms significantly impact daily activities, they are often not considered in clinical trials and disease management [12, 75]. It should be noted that some effective antihistamines are not available in pediatric doses or liquid formulations, or have not been sufficiently tested in young children [40].

There are multiple other uses for oral antihistamines in children. Well established ones include allergic conjunctivitis and urticaria. Others that have experienced variably positive recommendations are atopic dermatitis, mosquito bite reactions, Wells’ syndrome, and non-allergic rhinitis [76]. Because of its superior safety profile, other indications for the use of fexofenadine in children should be investigated. Furthermore, OTC second-generation antihistamines are recommended for the treatment of chronic urticaria in adults at doses two to four times the recommended label dose in Europe and US guidelines (off label), though these doses remain to be tested in children [77–80]. Furthermore, the efficacy of different fexofenadine dosing frequencies has not been investigated in children. Although currently recommended to be administered twice a day, there is evidence to suggest that once daily dosing may not only be clinically efficacious, but may also improve medication adherence in the pediatric population.

## Conclusions

AR is one of the most common pediatric chronic diseases; however, it is often under diagnosed and under treated. Although not life-threatening, the profound impact of AR is often underestimated, which is unfortunate given the detrimental impact on comorbidities and QoL, including school performance and social activities [2, 3].

First-generation antihistamines have been available OTC for nearly 80 years and, as a result, have been, and continue to be frequently used in children [32]. However, unguided use of first-generation OTC antihistamines can often lead to a range of mild to severe CNS side effects, accidental overdose and even death due to the associated anticholinergic activity in children, even at recommended doses [1, 2]. As such, treatment guidelines now recommend the use of non-sedating, second-generation antihistamines, particularly in children [2]. Fexofenadine, a second-generation antihistamine, not only lacks the sedative and cardiac adverse effects displayed by first-generation antihistamines, but has been rated superior to other antihistamines with respect to AR symptom management [12, 27, 32, 81]. Overall, fexofenadine is associated with greater treatment satisfaction in children with respect to efficacy, tolerability and impact on sleep and school performance [61].

## Supplementary Information

**Additional file 1: Table S1.** Adverse events reported for fexofenadine and placebo in clinical trials. **Table S2.** Evidence of efficacy of fexofenadine for the treatment of pediatric AR.

## Data Availability

Not applicable.

## Abbreviations

AR: Allergic rhinitis
BID: Twice daily
CNS: Central nervous system
GI: Gastrointestinal
H_1_: Histamine
IgE: Immunoglobulin E
MSC: Major symptoms complex
NSS: Nasal symptom score
OTC: Over-the-counter
PR interval: Time from the onset of the P wave to the start of the QRS complex
QD: Once daily
QoL: Quality of life
QTc: Corrected QT interval
REM: Rapid eye movement
TNSS: Total nasal symptom score
TSS: Total symptom score
URI: Upper respiratory tract infection
