# Correction: Fexofenadine: review of safety, efficacy and unmet needs in children with allergic rhinitis

**DOI:** 10.1186/s13223-022-00754-3

**Published:** 2022-12-27

**Authors:** Eli O. Meltzer, Nelson Augusto Rosario, Hugo Van Bever, Luiz Lucio

**Affiliations:** 1 Department of Pediatrics, Division of Allergy and Immunology, University of California, La Jolla, San Diego, CA USA; 2 Departamento de Pediatria, Universidade Federal Do Parana, Curitiba, PR Brazil; 3 Department of Pediatrics, Division of Rheumatology, Immunology, Allergy, Yong Loo Lin School of Medicine, National University of Singapore, Singapore, Singapore; 4 Medical Department, Sanofi Consumer Healthcare, AI, Traira 456, Santana de Parnaiba, SP 06540 365 Brazil

**Correction to: Allergy, Asthma & Clinical Immunology 17: 113 (2021)** https://doi.org/10.1186/s13223-021-00614-6

Unfortunately, there is an error in a statement and two references were incorrectly cited. We would like to update the statement and the references as detailed below.

Corrections in original article [1]:

1. Reference 55, Godse KV, Nadkarni NJ, Jani G, Ghate S. Fexofenadine in higher doses in chronic spontaneous urticaria. Indian Dermatol Online J. 2010;1(1):45–6. https://doi.org/10.4103/2229-5178.73262, does not support the statement. This reference has been replaced with the following: Estelle F & Simons R. The value of a broad therapeutic index for antihistamines. Adv Studies Med. 2002;2(24):872–876.
2. Reference 56, Fexofenadine: https//www.drugs.com/pro/fexofenadine.html, also does not support the statement. This reference has been replaced with the following: Howarth PH. The concept of the therapeutic window in the choice of H1-receptor antagonist. Adv Studies Med. 2004;4(7):S508–512.
3. To ensure that the statement is scientifically correct and aligns with the updated references 55 and 56, the following sentence ‘The established therapeutic range for fexofenadine in adults and children over 12 years is 20–240 mg’ has been replaced with ‘**Fexofenadine has demonstrated a wide therapeutic index in adults and children over 12 years, with 20 mg twice daily the minimally effective dose, and no sedation or cardiac toxicity noted at 690 mg twice daily for 28 consecutive days**’

Notes:

1. The original references did not mention the therapeutic index for fexofenadine and were cited in error. The replacement references support the edited statement.
2. The original statement was not scientifically accurate as it did not reflect the actual therapeutic index for fexofenadine. The suggested edits to the statement are supported by the replacement references.
