# Supplementary material for: Fexofenadine: review of safety, efficacy and unmet needs in children with allergic rhinitis
Source: Allergy Asthma Clin Immunol. 2021 Nov 2;17:113. doi: 10.1186/s13223-021-00614-6 (PMC8561980; doi:10.1186/s13223-021-00614-6)
Supplement: Supplementary file 1 — Additional file 1: Table S1. Adverse events reported for fexofenadine and placebo in clinical trials. Table S2. Evidence of efficacy of fexofenadine for the treatment of pediatric AR. [file 13223_2021_614_MOESM1_ESM.docx]

**Journal name:** *Allergy, Asthma & Clinical Immunology*

**Title: Fexofenadine: Review of safety, efficacy and unmet needs in children with allergic rhinitis**

**Authors: Meltzer, Eli O^1^ M.D., Rosario, Nelson Augusto^2^ M.D., Van Bever, Hugo^3^ M.D., ^*^Lucio, Luiz^4^ M.D.**

**Corresponding author:** Dr Luiz Lucio

*Medical Department, Sanofi Consumer Healthcare, São Paulo, Brazil^*^*

^*^Current address: AI, Traira 456, Santana de Parnaiba-SP, Brazil, 06540 365

Tel: +55 11 99461 3801; E-mail: luaglucio@gmail.com

**^*^Affiliation correct at the time of the study**

**Supplementary Table 1: Adverse events reported for fexofenadine and placebo in clinical trials**

| **Reference** | **Number of patients reporting adverse events (fexofenadine/total population)** | | | |
| --- | --- | --- | --- | --- |
|  | **Number of patients** | **Age (years)** | **Fexofenadine dose and frequency** | **Most common adverse events, n** |
| Hampel et al.[1] | 392 | 6 months – 2 years | 15, 30 mg BID for 1 week | Vomiting (18/193), diarrhoea (10/193), otitis media (10/193), rash (7/193), URI (7/193), fever (7/193) and cough increased (6/193) |
| NCT01244217  [2] | 100 | 6 months – 11 years | 15–30 mg BID for 4–12 weeks | Nasopharyngitis (4/7, 26/51 and 22/51, for those aged <2, 2 to<7 and >=7, respectively) |
| Milgrom et al.[3] | 453 | 2–5 | 30 mg BID for 2 weeks | Fever (13/222), vomiting (11/222), accidental injury (10/222), cough increased (10/222), URI (9/222), rhinitis (9/222), infection (8/222), otitis media (8/222), headache (7/222), GI pain (6/222) and diarrhoea (5/222) |
| Wahn et al.[4] | 85/88 | 6–11 | 30 mg BID for 2 weeks | Headache (23/464), epistaxis (7/464), URI (7/464), pharyngitis (6/464), sinusitis (6/464), nausea (5/464) and rash (5/464) |
| Ngamphaiboon et al.[5] | 88 | 6–11 | 30 mg BID for 2 weeks | Headache (6/88) |
| Meltzer et al.[6] | 1810 | 6–11 | 15, 30, 60 mg BID for 2 weeks | Headache (80/1110), URI (24/1110), cough increased (19/1110), sore throat (19/1110), accidental injury (18/1110), epistaxis (17/1110), infection (16/1110), GI pain (15/1110), fever (14/1110), rash (13/1110), pain (12/1110) and nausea (11/1110) |
| Graft et al.[7] | 875 | 6–11 | 15, 30, 60 mg BID for 2 weeks | Headache (53/646), URI (23/646), pharyngitis (21/646), injury accident (19/646), coughing (16/646), abdominal pain (15/646) and fever (13/646) |

BID, twice daily; GI, gastrointestinal; URI, upper respiratory tract infection.

**Supplementary Table 2: Evidence of efficacy of fexofenadine for the treatment of pediatric AR**

| **Type of study** | **Year** | **Study length** | **Age of patients (years)** | **Number of patients** | **Fexofenadine dose and frequency** | **Outcome** | **Reference** |
| --- | --- | --- | --- | --- | --- | --- | --- |
| Non-randomized, open label trial | 2010 | 12 weeks | 6 months – 11 years | 100 | 15–30 mg BID | Mean TSS decrease of -1.78 | [2] |
| Interventional | 2002 | 2 weeks | 6–11 | 264 | 30 mg QD | Fexofenadine resulted in complete symptom relief in  74.2% and marked relief reported in  24.6% of patients | [8] |
| Double-blind, randomized study | 2003 | 2 weeks | 6–11 | 935 | 30 mg BID | Mean change in PM-reflective scores was 1.94  AM-reflective TSS -1.67 | [4] |
| Pooled analysis of double-blind, randomized trials | 2004 | 2 weeks | 6–11 | 1810 | 15, 30, 60 mg BID | Mean TSS decrease of -1.75 | [6] |

BID, twice daily; MSC, major symptoms complex; NSS, nasal symptom score; TNSS, total nasal symptom score; TSS, total symptom score; QD, once daily.

**References**

1. Hampel, F.C., B. Kittner and J.H. van Bavel, *Safety and tolerability of fexofenadine hydrochloride, 15 and 30 mg, twice daily in children aged 6 months to 2 years with allergic rhinitis.* Ann Allergy Asthma Immunol, 2007. **99**(6): p. 549-54 DOI: 10.1016/S1081-1206(10)60385-7.

2. Safety, Efficacy and Pharmacokinetic Study of Allegra in Pediatric Patients With Perennial Allergic Rhinitis (PAR) [Available from: https://ClinicalTrials.gov/show/NCT01244217.

3. Milgrom, H., et al., Safety and tolerability of fexofenadine for the treatment of allergic rhinitis in children 2 to 5 years old. Ann Allergy Asthma Immunol, 2007. 99(4): p. 358-63 DOI: 10.1016/S1081-1206(10)60553-4.

4. Wahn, U., et al., Fexofenadine is efficacious and safe in children (aged 6-11 years) with seasonal allergic rhinitis. J Allergy Clin Immunol, 2003. 111(4): p. 763-9 DOI: 10.1067/mai.2003.1384.

5. Ngamphaiboon, J., et al., The efficacy and safety of 30 mg fexofenadine HCl bid in pediatric patients with allergic rhinitis. Asian Pac J Allergy Immunol, 2005. 23(4): p. 169-74

6. Meltzer, E.O., et al., Safety and efficacy of oral fexofenadine in children with seasonal allergic rhinitis--a pooled analysis of three studies. Pediatr Allergy Immunol, 2004. 15(3): p. 253-60 DOI: 10.1111/j.1399-3038.2004.00167.x.

7. Graft, D.F., et al., Safety of fexofenadine in children treated for seasonal allergic rhinitis. Ann Allergy Asthma Immunol, 2001. 87(1): p. 22-6 DOI: 10.1016/S1081-1206(10)62317-4.

8. Fexofenadine Study On Filipino Children for The Relief of Perennial And Intermittent Allergic Rhinitis [Available from: https://ClinicalTrials.gov/show/NCT00741897.
